# Contribution of Autonomic Reflexes to the Hyperadrenergic State in Heart Failure

**DOI:** 10.3389/fnins.2017.00162

**Published:** 2017-03-30

**Authors:** Edgar Toschi-Dias, Maria Urbana P. B. Rondon, Chiara Cogliati, Nazareno Paolocci, Eleonora Tobaldini, Nicola Montano

**Affiliations:** ^1^Heart Institute (InCor) do Hospital das Clínicas da Faculdade de Medicina da Universidade de São PauloSão Paulo, Brazil; ^2^Department of Internal Medicine, Fondazione IRCCS Ca' Granda Ospedale Maggiore PoliclinicoMilan, Italy; ^3^School of Physical Education and Sports, University of São PauloSão Paulo, Brazil; ^4^Medicina ad Indirizzo Fisiopatologico, ASST Fatebenefratelli SaccoMilan, Italy; ^5^Division of Cardiology, Department of Medicine, Johns Hopkins Medical InstitutionsBaltimore, MD, USA; ^6^Dipartimento di Medicina Sperimentale, Universita' degli Studi di PerugiaPerugia, Italy; ^7^Dipartimento di Dipartimento Scienze cliniche e di comunità, Università degli Studi di MilanoMilan, Italy

**Keywords:** heart failure, autonomic nervous system, sympathetic nerve activity, cardiovascular variability

## Abstract

Heart failure (HF) is a complex syndrome representing the clinical endpoint of many cardiovascular diseases of different etiology. Given its prevalence, incidence and social impact, a better understanding of HF pathophysiology is paramount to implement more effective anti-HF therapies. Based on left ventricle (LV) performance, HF is currently classified as follows: (1) with reduced ejection fraction (HFrEF); (2) with mid-range EF (HFmrEF); and (3) with preserved EF (HFpEF). A central tenet of HFrEF pathophysiology is adrenergic hyperactivity, featuring increased sympathetic nerve discharge and a progressive loss of rhythmical sympathetic oscillations. The role of reflex mechanisms in sustaining adrenergic abnormalities during HFrEF is increasingly well appreciated and delineated. However, the same cannot be said for patients affected by HFpEF or HFmrEF, whom also present with autonomic dysfunction. Neural mechanisms of cardiovascular regulation act as “controller units,” detecting and adjusting for changes in arterial blood pressure, blood volume, and arterial concentrations of oxygen, carbon dioxide and pH, as well as for humoral factors eventually released after myocardial (or other tissue) ischemia. They do so on a beat-to-beat basis. The central dynamic integration of all these afferent signals ensures homeostasis, at rest and during states of physiological or pathophysiological stress. Thus, the net result of information gathered by each controller unit is transmitted by the autonomic branch using two different codes: *intensity* and *rhythm* of sympathetic discharges. The main scope of the present article is to (i) review the key neural mechanisms involved in cardiovascular regulation; (ii) discuss how their dysfunction accounts for the hyperadrenergic state present in certain forms of HF; and (iii) summarize how sympathetic efferent traffic reveal central integration among autonomic mechanisms under physiological and pathological conditions, with a special emphasis on pathophysiological characteristics of HF.

“*Most of our faculties lie dormant because they can rely upon Habit, which knows what there is to be done and has no need of their services.”*

**Marcel Proust**

## Introduction

Almost all cardiovascular disease conditions eventually culminate in heart failure (HF), a complex and multifactorial syndrome that remains the main cause of morbidity and mortality worldwide (Ponikowski et al., 2016). HF stems from progressive structural and functional deterioration of the myocardium, due to chronic hemodynamic stress, whose initial trigger could be of ischemic or non-ischemic origin (Brede et al., 2002; Triposkiadis et al., 2009). Regardless of its etiology, neurohumoral activation represents a major contributor of HF pathophysiology. The neurohumoral activation arises as a compensatory response in order to adjust cardiac performance in the face of increased workload. However, persistent hemodynamic stress results in chronic release of neurohormones, particularly from sympathetic efferents and the adrenal medulla (Lymperopoulos et al., 2013), which play a major role in the progressive deterioration of cardiac function during HF (Brede et al., 2002). A hyperadrenergic state can be found in clinical conditions such as hypertension (Miyajima et al., 1991), coronary artery disease and myocardial infarction (Graham et al., 2002). As mentioned above, presence of a chronic hyperadrenergic state paves the way for HF (Notarius et al., 2007), yet also serves as a major index in the prognosis of HF (Barretto et al., 2009) and as a central therapeutic target.

Depending on the status of left ventricular (LV) performance, HF is currently classified in three clinically distinct syndromes (Ponikowski et al., 2016). Patients exhibiting signs and/or symptoms of HF that display LV ejection fraction (LVEF) <40% are categorized as HF patients with reduced ejection fraction (HFrEF) (Ponikowski et al., 2016). HF patients with preserved systolic function (LVEF >50%), but with diastolic dysfunction (LV end-diastolic volume index <97 ml/m^2^) are now defined as HF patients with preserved ejection fraction (HFpEF) (Ponikowski et al., 2016; van Heerebeek and Paulus, 2016). Finally, based on more recent international guidelines on HF, a third group of HF subjects has now been identified. Those patients who display symptoms of HF with moderately reduced systolic function (LVEF between 40 and 50%) are now classified as HF with mid-range EF (HFmrEF) (Ponikowski et al., 2016).

Clinically, dyspnea and exercise intolerance are fundamental symptoms of HF. They arise at the onset of disease, and progress according to the severity of cardiac dysfunction (Ponikowski et al., 2016). Complex neuro-hormonal alterations are at the foundation of these symptoms, and act in concert with increased pulmonary venous pressure, decreased peripheral blood flow, endothelial dysfunction and skeletal muscle abnormalities (Floras and Ponikowski, 2015). It is well established that perturbed reflex mechanisms sustain sympathetic hyperactivity during HFrEF. However, substantial efforts are needed to identify changes in neural-cardiovascular regulatory pathways in HFpEF patients. Indeed, these patients also have prominent autonomic dysfunction (Verloop et al., 2015). However, little is known about the role of neural mechanisms that govern the amplitude or frequency of bursts of autonomic activity, or the pattern of active fiber discharge. Likewise, the central pathways that affect sympathetic burst generation in HFmrEF and HFpEF patients are not fully understood.

In this article, we will review and discuss old and new acquisitions on (i) the principal neural/reflex mechanisms accounting for cardiovascular homeostasis, (ii) how perturbations in these “controller units” account for the hyperadrenergic state characterizing HF; and (iii) how these mechanisms interact under physiological conditions as well as in subjects affected by HF of different categories.

## Hyperadrenergic state in heart failure

Under physiological conditions, several regulatory mechanisms work in concert in order to modulate the activity of the autonomic nervous system. By regulating heart rate, blood pressure and peripheral blood flow, the autonomic nervous system dynamically adjusts the functions of the cardiovascular system to ensure adequate levels of cardiac output will meet the perfusion and metabolic requirements of peripheral organ systems (Salman, 2016).

The past few decades have seen a growing research interest in the studying of neural mechanisms of cardiovascular regulation. This investigative attention was, and still is, justified by the recognition that these autonomic reflex controls are critical in the detection and correction of spontaneous changes in arterial blood pressure, thoracic blood volumes and pressures, humoral factors produced during myocardial ischemia and changes in arterial concentration of oxygen, carbon dioxide and pH. These reflexes are indeed essential to maintain whole-body homeostasis at rest (Salman, 2016), or during ordinary physiological events, such as, (i) changes in posture (i.e., clinostatic vs. orthostatic; Montano et al., 1994), (ii) alterations in the sleep-wake cycle (Narkiewicz et al., 1998; Tobaldini et al., 2017), (iii) in response to exercise (Negrão et al., 2001), or (iv) during emotional stress (Durocher et al., 2011).

However, during disease states affecting the cardiovascular system, the disturbance in regulatory mechanisms may cause autonomic dysfunction, in one or more of these neural/reflex mechanisms, ultimately resulting in an overall sympathetic hyperactivation and/or vagal impairment. When chronically sustained, the resulting autonomic imbalance triggers a complex “vicious cycle” that contributes to the onset of cardiovascular disease (Floras and Ponikowski, 2015).

**Figure 1:**
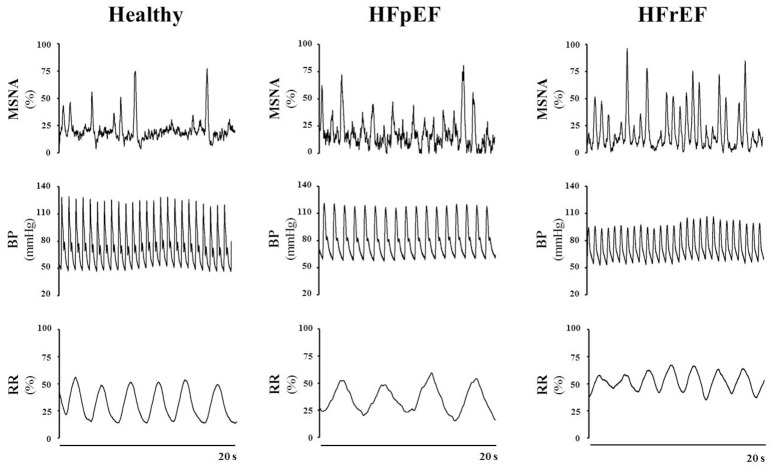
**Representative tracings of muscle sympathetic nerve activity (MSNA), blood pressure (BP) and respiration rate (RR) in a healthy subject, a patient with heart failure with preserved ejection fraction (HFpEF) and a patient with heart failure with reduced ejection fraction (HFrEF), at rest**. Note the difference in the discharge patterns of the sympathetic outflow between healthy control subjects vs. HFpEF vs. HFrEF patients. Representative tracings of MSNA, BP and RR are unpublished material.

As mentioned above, a hallmark of HF is the hyperadrenergic state, characterized by an exacerbated sympathetic nerve discharge and a progressive loss of rhythmical sympathetic oscillation (van de Borne et al., 1997; Barretto et al., 2009) (Figure 1). In particular, patients with HFrEF, display eccentric LV remodeling and systolic dysfunction, yet cardiac output and tissue perfusion pressures are maintained. The latter two are maintained, at least initially, by the sustained activation of the sympathetic nervous system (SNS) and the renin-angiotensin aldosterone system (Roig et al., 2000; Notarius et al., 2007). Conversely, HFpEF patients exhibit LV concentric hypertrophy, interstitial fibrosis and capillary rarefaction, with the main functional defect contributing to diastolic dysfunction is impaired cardiomyocyte relaxation (Paulus and Tschope, 2013). Sympathetic overdrive has been documented in these patients as well. Verloop and colleagues recently concluded that the current availability of knowledge does not permit a distinguishment on whether enhanced sympathetic activity results in HFpEF, or HFpEF results in enhanced sympathetic activity (Verloop et al., 2015). Moreover, whether patients with HFmrEF present with a hyperadrenergic state, and the eventual magnitude of this alteration, remains to be documented clinically.

### Arterial baroreflex control

**Figure 2:**
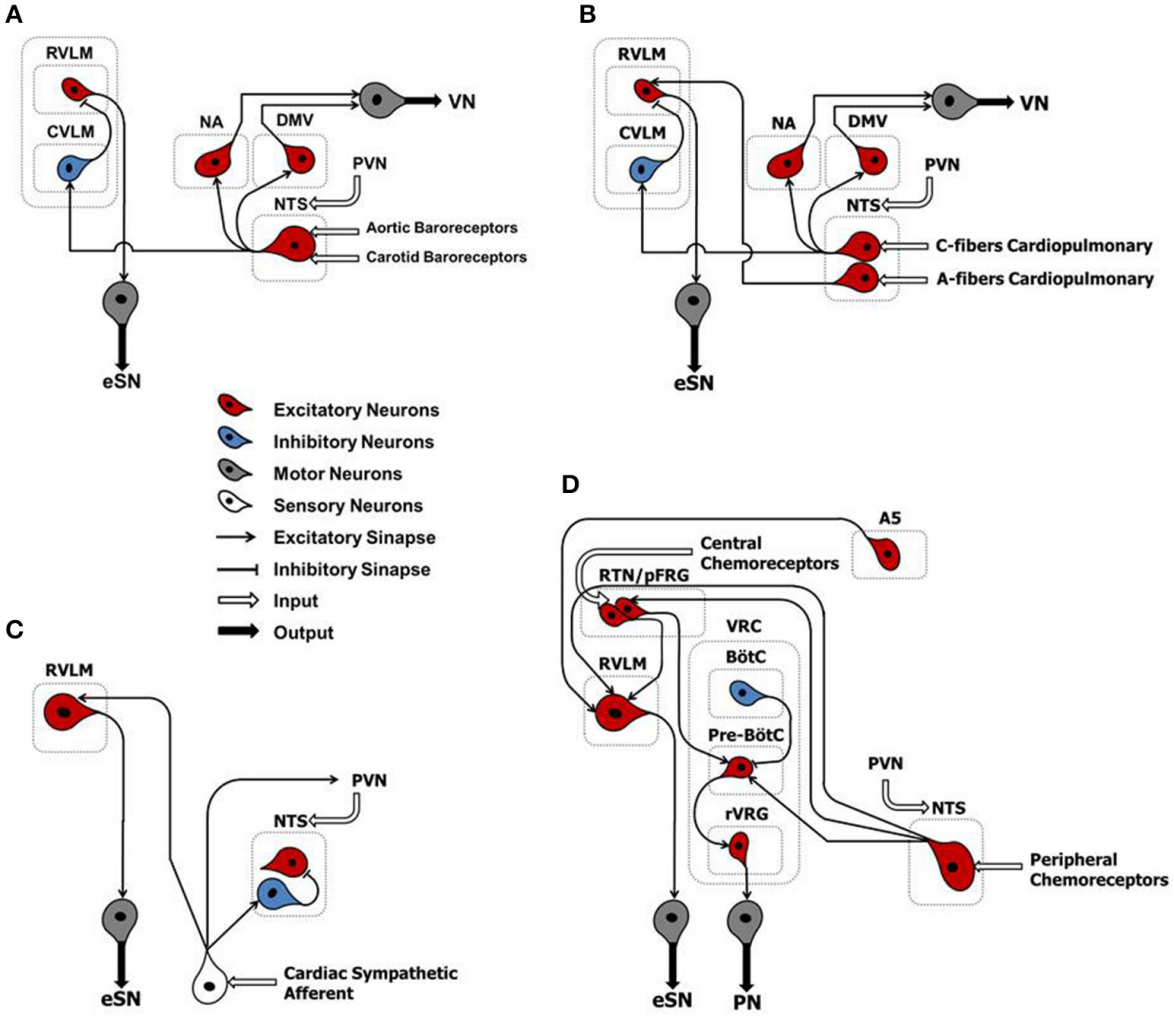
**Classical schematic diagram of arterial baroreflex control (A)**, cardiopulmonary reflex **(B)**, cardiac sympathetic afferent reflex **(C)**, and arterial chemoreflex **(D)**. NTS, nucleus tractus solitarii; NA, nucleus ambiguus; DMV, dorsal motor nucleus of the vagus; CVLM, caudal ventrolateral medulla; RVLM, rostral ventrolateral medulla; PVN, paraventricular nucleus of the hypothalamus; A5, noradrenergic neurons of the ventrolateral pons; RTN/pFRG, retrotrapezoid nucleus/parafacial respiratory group; VRC, ventral respiratory column; BötC, Bötzinger complex; pre-BötC, pre-Bötzinger complex; Rvrg, rostral ventral respiratory group; eSN, efferent sympathetic nerve; PN, phrenic nerve; VN, vagus nerve.

Arterial baroreflex (ABR) control is an integrated negative-feedback system whose main role is to stabilize arterial blood pressure in response to changes of circulatory homeostasis, in the short-term (Salman, 2016). Stretch-sensitive receptors located in specialized regions of the aortic arch and carotid sinus are innervated by branches of the IX and X cranial nerves. They discharge in response to an increase in arterial pressure. The central projections of baroreceptor afferents terminate primarily in the intermediate portion of the nucleus tractus solitarii (NTS) that form asymmetric (excitatory) synaptic contacts through glutamatergic receptors with caudal ventrolateral medulla (CVLM) (Figure 2A). In turn, CVLM efferent terminals form symmetric (inhibitory) synaptic contacts through GABAergic receptors with reticulospinal and adrenergic neurons of the rostral ventrolateral medulla (RVLM), thus inhibiting sympathetic outflow (Pilowsky and Goodchild, 2002) (Figure 2A). In parallel, other second-order NTS neurons form asymmetric synaptic contacts with the dorsal motor nucleus of the vagus nerve, maintaining an excitatory influence upon preganglionic parasympathetic neurons (Pilowsky and Goodchild, 2002) (Figure 2A).

Thus, an elevation in arterial blood pressure triggers an increase in the baroreceptor firing rate to medullary nuclei of central integration, leading to an increased discharge of vagal efferent fibers and a decreased sympathetic outflow. The physiological response of these combined effects results in bradycardia and a decrease in cardiac contractility, peripheral vascular resistance, and venous return, all acting to counter the rise in arterial blood pressure (Kirchheim, 1976; Tank et al., 2005). Conversely, a decrease in arterial blood pressure results in a reduced baroreceptor firing rate. Culminating in sympathetic excitation and parasympathetic withdrawal, thus leading to tachycardia, increased cardiac inotropy, vascular resistance, and venous return (Kirchheim, 1976; Tank et al., 2005).

During each beat, the ABR control is the main modulator of the activity of the autonomic nervous system during short-term variation of arterial pressure (Pagani and Malliani, 2000; Tank et al., 2005). Classical studies in humans and animals have clearly proved that the hyperadrenergic state of HFrEF is accompanied by blunting of ABR (Ferguson et al., 1992; Grassi et al., 2001) (Table 1).

**Table 1:** **Autonomic reflex control of sympathetic nerve activity in patients with heart failure**.

	**HFrEF**	**HFpEF**
Arterial Baroreflex	Blunted	–
Cardiopulmonary Reflex	Paradoxical	–
Cardiac Sympathetic Afferents Reflex	Exacerbated^a^	–
Arterial Chemoreflex	Exacerbated	Exacerbated^a^

*HFrEF, heart failure with reduced ejection fraction; HFmrEF, heart failure with mid-range ejection fraction; HFpEF, heart failure with preserved ejection fraction*.

a *Only in animal model*.

As we learned before, in HFrEF patients that display eccentric remodeling and systolic dysfunction, cardiac output and tissue perfusion pressure are maintained via sustained neurohormonal activation (Zucker et al., 2004). In the heart, chronic sympathetic activation promotes myocardial hypertrophy and vascular remodeling (Zucker et al., 2004; Floras and Ponikowski, 2015). Moreover, vascular remodeling could play an important role in the maintenance of sympathetic drive in HF, by affecting the afferent branch of ABR. In rats with ischemia-induced HF, the gain of afferent aortic depressor nerve is reduced (Rondon et al., 2006). Thus, arterial stiffness could impair the mechanical transduction of baroreceptors, therefore affecting afferent traffic.

No studies, to our knowledge, have directly examined the impact of ABR control on sympathetic nerve activity in patients with HFpEF and HFmrEF (Table 1). However, data from an experimental study suggest that baroreflex dysfunction of heart rate is associated with cardiac diastolic dysfunction in rats, independent of other present risk factors (Mostarda et al., 2011). Moreover, patients with HFpEF are generally older, more often female, and have a high prevalence of comorbidities, such as chronic hypertension, obesity, metabolic syndrome and type 2 diabetes mellitus (Paulus and Tschope, 2013; van Heerebeek and Paulus, 2016). All these conditions have been associated, in part, with baroreflex dysfunction (Matsukawa et al., 1991, 1994; Trombetta et al., 2010; Holwerda et al., 2016). In particular, an association exists between hypertension and vascular injury (Dao et al., 2005), affecting the elastic properties of the large vessels (Humphrey et al., 2016; Smulyan et al., 2016). This, in turn, could impact the efficiency of ABR control in patients with HFpEF. Therefore, myocardial remodeling and arterial stiffness can affect ABR arc function through different pathophysiological mechanisms, leading to sympathetic hyperactivation both in HFrEF and HFpEF patients.

### Cardiopulmonary reflex

The cardiopulmonary reflex arc is liable for detecting filling pressure in low-pressure cardiac chambers. In doing so, it contributes to the control of volemic conditions through the interaction between negative- and positive-feedback mechanisms (Salman, 2016). Several receptor subtypes, located in the heart and lungs, contribute to this reflex arc. They are mechano-sensitive and closely linked to neuronal pathways via overlapping brainstem networks. Whereas, most of these receptors are innervated by unmyelinated afferents of the vagus nerve (Type C) and are activated at higher intensities, a smaller portion is made of myelinated fibers of the vagus nerve (Type A) and are activated at lower intensities (Salman, 2016). Cardiopulmonary vagal afferents are mainly involved in neurohumoral regulation of systemic blood volume. When stimulated, they convey information to medullary nuclei of central integration (i.e., NTS) that form a trisynaptic intramedullary pathway similar to that described for the ABR (Salman, 2016) (Figure 2B).

Firstly, fluctuations of lower intensities activate sensory afferent fibers (Type A) located in the walls of the atria and in the atrial-caval junction, determining tachycardia through sympathetic activation. Atrial stretch results in the release of atrial natriuretic peptide by atrial myocytes, which act to increase renal blood flow and Na^+^ diuresis, reducing volume and keeping cardiac output relatively constant during the increase of venous return (Salman, 2016). In stark contrast, fluctuations of higher intensities activate Type C sensory afferent fibers located in cardiac chambers, strengthening and enhancing the action of ABR by means of similar negative-feedback mechanisms. However, during hypovolemia, the neurohormonal response acts to reduce heart rate (bradycardia) and urinary volume in order to correct the initial drop in venous return (Salman, 2016).

In 1990, Seals and colleagues observed that during controlled tidal breathing (30% of inspiratory capacity at 12 breath/min), approximately 65% of the sympathetic bursts occur during the expiratory phase, suggesting an influence of breathing on sympathetic outflow (Seals et al., 1990). In turn, increasing the depth of breathing (e.g., 70% of inspiratory capacity at 12 breath/min) has great influence on the within-breath modulation of muscle sympathetic nerve activity (MSNA), producing near complete sympathoinhibition from onset-mid inspiration to early-mid expiration (Seals et al., 1990).

The effects of cardiopulmonary reflex activation and deactivation on sympathetic outflow has been evaluated in humans via MSNA recording during lower body negative/positive pressures. Previously demonstrated, deactivation of cardiopulmonary reflex during application of non-hypotensive lower body negative pressure (LBNP) showed an increase in the sympathetic efferent traffic in healthy subjects (Millar et al., 2015). Conversely, activation of cardiopulmonary reflex during application of non-hypertensive lower body positive pressure (LBPP) was associated with a decrease in sympathetic activity in healthy subjects (Millar et al., 2015).

Alterations of the autonomic neural reflex responses due to changes in cardiopulmonary loading conditions have been well documented in HFrEF patients (Azevedo et al., 2000; Floras and Ponikowski, 2015). Recently, Millar and colleagues reported that despite a preserved response of MSNA during deactivation of the cardiopulmonary reflex arc, non-hypertensive LBPP does not change the burst frequency of sympathetic outflow in patients with HFrEF (Millar et al., 2015). This paradoxical sympathetic activation in response to increasing filling pressure suggests that cardiopulmonary reflex contributes to the complex generation of autonomic dysregulation of HFrEF. Similar evidence in HFpEF and HFmrEF patients is lacking (Table 1).

### Cardiac sympathetic afferent reflex

The cardiac sympathetic afferent reflex (CSAR) is a positive feedback mechanism involved in the central transmission of nociceptive information from the heart (Chen et al., 2015). The receptors of this reflex arc are the cardiac sympathetic afferent endings that innervate the superficial epicardial layers of the ventricles. Although the exact neural pathways of CSAR are not yet well understood, there is a growing consensus of opinion that the central projections of sympathetic afferents located in the dorsal root ganglia of the C8–T9 spinal segments (especially T2–T6) form excitatory synapses in the hypothalamic paraventricular nucleus (PVN), NTS and RVLM. These stations in turn centrally process the peripheral information and, when stimulated, induce an increase in sympathetic outflow (Chen et al., 2015) (Figure 2C). Thus, mechanical distension of the ventricles and/or endogenous humoral factors produced in myocardium during myocardial ischemia, such as bradykinin, adenosine and reactive oxygen species (ROS), can stimulate cardiac sympathetic afferents, thereby increasing the sympathetic outflow directed to the heart, arteries and kidneys (Chen et al., 2015). All these mechanisms take part in the excessive sympathetic outflow that characterizes HF (Malliani and Montano, 2002; Zhu et al., 2004; Wang et al., 2014) (Table 1). Furthermore, once overtly dilated, the heart tends to utilize more oxygen, owing to increased cardiac wall tension. This need renders the heart more prone to “relative ischemia,” a functional feature often observed in HF patients. In turn, this condition is accompanied by the release of endogenous substances such as bradykinin and adenosine that stimulate the ventricular sympathetic afferents endings, resulting in increased sympathetic outflow due to positive feedback mechanism. Furthermore, chronic myocardial stretch due to volume overload can cause an upregulation of the kallikrein-kinin system, followed by an increase in bradykinin content in the interstitial fluid that ultimately mediates mast cell infiltration and extracellular matrix loss (Wei et al., 2012).

In a clinical context, it is well known that patients with acute decompensated HF are very sensitive to volume overload because they have volume intolerance, regardless of the magnitude of residual LVEF. Moreover, during acute decompensated HF and pulmonary congestion, both arterial pressure and heart rate are frequently more elevated (De Luca et al., 2007). It is still unclear how the positive feedback mechanism mediated by CSAR would eventually prevail over the negative one mediated by the vagal afferents. However, there is an attractive corollary hypothesis to consider (Malliani and Montano, 2002). For example, afferent vagal fibers from the atria normally display a burst of impulses per cardiac cycle. In the case of Type A receptors, this event occurs in coincidence with atrial systole (Paintal, 1971). In central structures, rhythmic afferent bursts can prompt a sequence of excitatory and inhibitory postsynaptic potentials (Spyer, 1982). During volume loading, Type A atrial vagal receptors also discharge during diastole (Recordati et al., 1976), and this more continuous firing may blunt the capability of generating inhibitory postsynaptic potentials in the central circuitry. Similar changes in firing patterns may characterize the majority of vagal cardiopulmonary receptors, thus explaining their reduced efficacy in exerting an efficient reflex restraint on the sympathetic outflow (Malliani and Montano, 2002). Conversely, under normal conditions, sympathetic afferent fibers do not display bursts of impulses. Rather, at most, they exhibit a more sparse and spontaneous activity with one action potential per cardiac cycle (Malliani, 1982). Thus, independent of its rhythmic relationship with the cardiac cycle, an increased afferent sympathetic barrage would retain its capability of exciting the sympathetic outflow (Malliani and Montano, 2002).

### Arterial chemoreflex

In response to environmental challenges, the maintenance of homeostasis depends also on the integration between neural mechanisms that adjust arterial levels of oxygen (O_2_), carbon dioxide (CO_2_) and potential of hydrogen (pH). To this end, the arterial chemoreflex control comprises *central chemoreceptors* in the brainstem that respond to hypercapnia and respiratory acidosis; and *peripheral chemoreceptors* in the carotid bodies (located near the internal carotid arteries) that respond primarily to hypoxia (Guyenet, 2000; Barnett et al., 2017).

During hypercapnia and respiratory acidosis, chemosensitive neurons located in retrotrapezoid nucleus/parafacial respiratory group (RTN/pFRG) are stimulated, sending excitatory inputs to the pre-sympathetic neurons of the RVLM that form excitatory synaptic contacts with noradrenergic neurons (A5 group) in the ventrolateral pons (Barnett et al., 2017) (Figure 2D). In parallel, second-order neurons of RTN/pFRG send excitatory inputs to the pre-Bötzinger complex–a cluster of neurons responsible for generating the breathing rhythm located in the ventral respiratory column (VRC). Here, they form excitatory synaptic contacts with the rostral ventral respiratory group (rVRG) and project to motor neurons that give rise to the phrenic nerve (Barnett et al., 2017) (Figure 2D).

During hypoxia, the central projections of peripheral chemoreceptor afferents (cranial nerve IX) terminate primarily in the commissural portion of the NTS that, by means of second-order neurons, then form excitatory synapses with RVLM, RTN/pFRG and the pre-Bötzinger complex (Guyenet, 2000; Barnett et al., 2017) (Figure 2D).

Therefore, the stimulation of chemoreflex by hypoxia and/or hypercapnia induces a simultaneous increase of sympathetic outflow and ventilation, thus optimizing tissue perfusion and blood gas uptake/delivery (Guyenet, 2000; Barnett et al., 2017) (Figure 2D).

In HFrEF, reduction in organ perfusion due to reduced cardiac output, promotes changes in oxygen delivery, thereby impairing peripheral chemoreflex function in HFrEF patients. In turn, chemoreflex dysfunction contributes to sympathetic hyperactivity, hyperventilation and the associated breathing instability typically found in HFrEF subjects (Schultz et al., 2015). Accordingly, Di Vanna and collaborators demonstrated that the activity of muscle sympathetic nerves in response to central and peripheral chemoreceptor stimulation is exacerbated in patients with HFrEF (Di Vanna et al., 2007) (Table 1).

In addition, it has been postulated that oxidative stress (i.e., increased ROS emission mainly from enhanced NADPH oxidase activity) is also central in activating chemoreceptors of the carotid bodies (Morgan et al., 2016). Oxidative stress is known to activate the carotid body in HF (Schultz et al., 2015). We now know that both circulating and local tissue levels of the pro-oxidant angiotensin II peptide are elevated in HF (Li et al., 2006). Angiotensin II activates NADPH oxidase to enhance superoxide production, which in turn enhances the excitability of the carotid body glomus cells and central autonomic neurons via the AT1 receptor (Li et al., 2007). The angiotensin II-superoxide pathway augments the sensitivity of the carotid body chemoreceptors, at least in part, by inhibiting oxygen-sensitive potassium channels in the carotid body glomus cells (Schultz et al., 2015).

Regardless of the low cardiac output, diverse comorbidities such as hypertension (Touyz, 2004), diabetes (Henriksen et al., 2011), and obstructive sleep apnea (Lavie, 2015) are associated with increased oxidative stress that may lead to carotid body chemoreceptor dysfunction. These comorbidities are frequent in patients with HFpEF or HFmrEF, thus suggesting the presence of chemoreflex dysfunction in these patients. In fact, Toledo and colleagues recently reported that the central chemoreflex is enhanced in HFpEF (Toledo et al., 2017). These authors observed that the activation of the central chemoreflex pathway by hypercapnia exacerbates sympatho-vagal imbalance in HFpEF rats, and that sympathetic blockade with propranolol attenuates the response of cardiac sympathetic modulation. In addition, these authors showed that neuronal activation in the RVLM was increased in HFpEF rats compared with sham rats (Toledo et al., 2017). However, further studies are needed to better understand the mechanistic intricacies of these new findings.

Further to this, to the best of our knowledge, there are no studies testing sympathetic nerve activity in response to chemoreceptor stimulation in patients with HFpEF or HFmrEF.

### Central integration of autonomic reflexes

**Figure 3:**
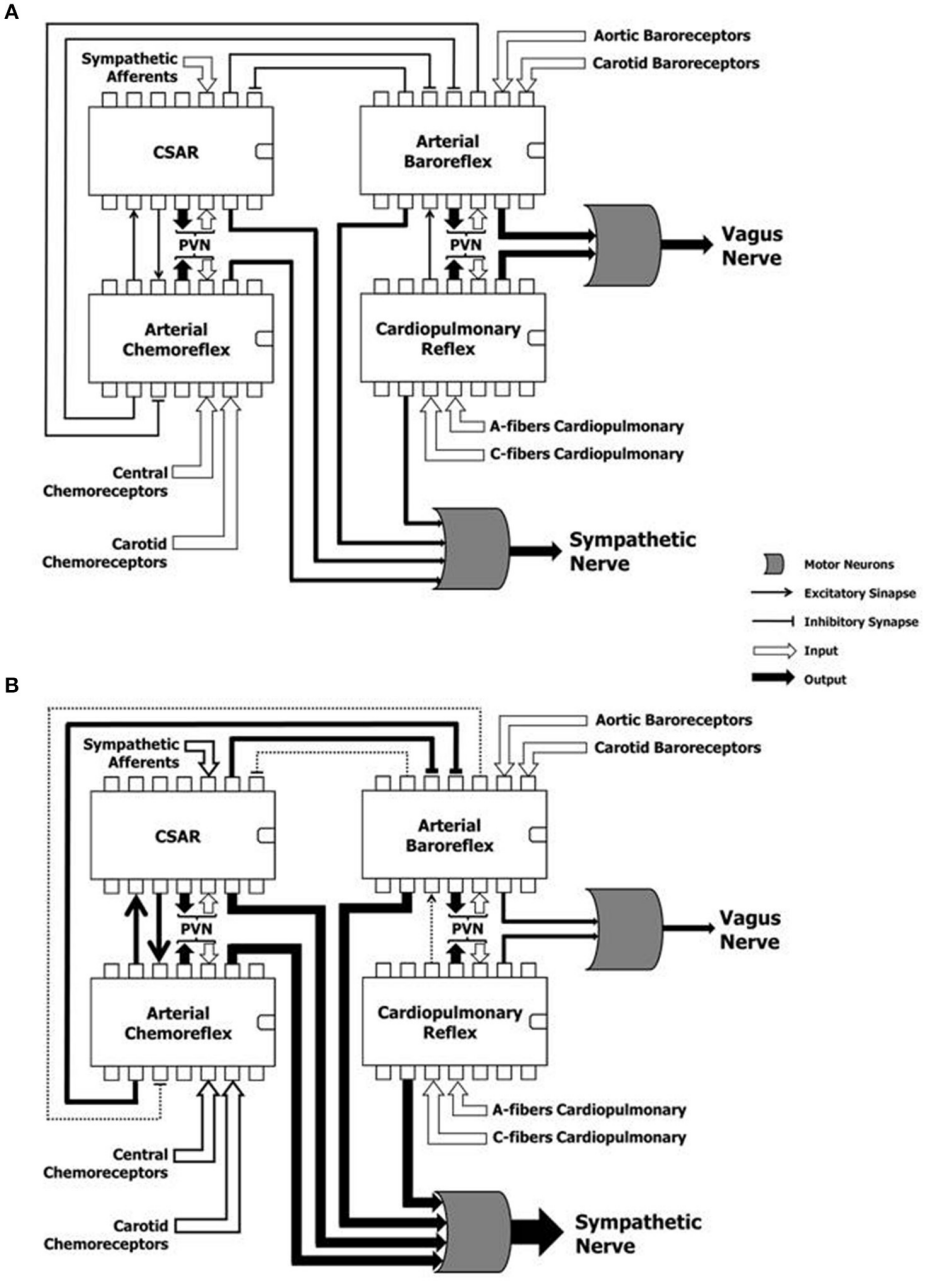
**Hypothetical representation of the reflex mechanisms considered as “controller units,” and their complex and dynamic interaction in a physiological condition (A)** and during heart failure **(B)**. CSAR, cardiac sympathetic afferents reflex; PVN, paraventricular nucleus of the hypothalamus; SN, sympathetic nerve; VN, vagus nerve. Note that the sustained hyperadrenergic state in patients with HF occurs due to the predominance of inputs of excitatory mechanisms on inhibitory mechanisms.

Autonomic reflexes are considered as distinct controllers units. However, multiple sites of interaction among these reflex arcs have been documented (Du and Chen, 2006; Chen et al., 2015). They are outlined in Figure 3. NTS is the first synaptic station of the afferents in the central nervous system. It plays a pivotal role in the modulation of the autonomic efferent activity directed to the cardiovascular system (Machado et al., 1997). In order to produce a proper autonomic response, information from several relay stations must be processed at the NTS level, where all the projections of many and complex neural networks are then organized in different hierarchical levels (Smith et al., 2009; Chen et al., 2015). For instance, activation of CSAR depresses ABR and enhances the arterial chemoreflex via central integration (Du and Chen, 2006; Chen et al., 2015) (Figure 3A). The afferent pathways of these reflex mechanisms project to the NTS, and numerous lines of investigation attests that baroreflex dysfunction of the MSNA is the consequence of and not the cause of the reflex sympathetic excitation seen in HF patients (Du and Chen, 2006; Despas et al., 2012) (Figure 3B). In this regard, an experimental study showed that electrical stimulation of the central end of the left cardiac sympathetic nerve blunts ABR sensitivity by 42% (Figure 3A), and this effect is abolished after intra-cerebroventricular injection of losartan (Gao et al., 2004). This evidence suggests that stimulation of cardiac sympathetic afferents reduces ABR sensitivity via central AT1 receptors (Gao et al., 2004).

AngII is expressed in the NTS, and one of the signaling mechanisms by which AT1 receptors influence the NTS neurons involves the production of ROS by NADPH oxidase. NADPH oxidase activation in turn enhances voltage-gated L-type Ca^2+^ currents in the medial NTS neurons, thereby contributing to the deleterious effects exerted by AngII in the cardiovascular system (Wang et al., 2004, 2006). As such, we cannot rule out a priori that AngII, via the AT1 receptor, plays a pivotal role in both inhibitory and excitatory reflexes in the central nervous system during HF. Accordingly, the augmented cardiac sympathetic afferent input can contribute to the exacerbated chemoreflex function in HF in a AT1 receptor-dependent manner in the NTS (Wang et al., 2008). Indeed, AT1 receptors in the NTS are upregulated, and blockade of these receptors normalized the exaggerated response of the chemoreflex/CSAR in HF (Wang et al., 2008).

In another study, Gan and colleagues observed that the CSAR is enhanced not only in HF rats, but also in rats with HF harboring bilateral vagotomy and ABR denervation (Gan et al., 2011). However, vagotomy and baroreceptor denervation augmented basal and AngII-stimulated CSAR response in the PVN (Gan et al., 2011). This study revealed that the activity of arterial baroreceptor and vagal afferents inhibit the CSAR (Figure 3B), while enhancing the CSAR discharge in response to AngII in the PVN of HF rats (Gan et al., 2011).

When the interaction between the chemoreflex and ABR is concerned (Figure 3A), we know that isocapnic hyperventilation (i.e., ventilation > 35 l/min at 15 breath/min) stimulates the chemoreflex and blunts ABR gain (van de Borne et al., 2000). On the other hand, the cardiovascular effect of chemoreflex stimulation also encompasses a rise in arterial blood pressure, which in turn, stimulates the arterial baroreceptors, while blunting the chemoreflex response (Somers et al., 1991) (Figure 3A).

Interestingly, Despas and colleagues have shown that elevated chemosensitivity attenuated ABR control of sympathetic nerve activity, and that deactivation of carotid chemoreceptors increased the ABR gain in patients with HFrEF (Despas et al., 2012) (Figure 3B). Together, this evidence suggests that the chemoreflex-related rise in sympathetic activity is not only mediated by an oxygen sensing mechanism, but also indirectly (i.e., through the involvement of ABR in patients with HFrEF; Despas et al., 2012). In stark contrast, the central integration among autonomic reflexes in patients with HFpEF and HFmrEF remains to be decodified.

## Questions to ponder for future studies

The relevance of the role and the modalities by which altered autonomic reflexes contribute to sympathetic hyperactivation in HFrEF are becoming increasingly clear. In contrast, little knowledge is currently available regarding this aspect in subjects with HFpEF or HFmrEF. Therefore, the new HF classification is likely to bring to the table additional, important questions concerning the role of autonomic control/reflexes in HF onset and progression. Notwithstanding, there are still major, general questions that pertain to the role of autonomic reflexes in HF pathophysiology, irrespective of its etiology or clinical classification. First and foremost, it is still unknown what is the cutoff and saturation point of the autonomic nervous system in the effector organ. As mentioned earlier, the information emanating from the central integration of many reflex mechanisms is transmitted by the autonomic fibers using *intensity* and *rhythm* of firing as codes. Importantly, certain clinical conditions can lead to the collapse of the autonomic nervous system and to the loss of rhythmicity of cardiovascular variability (Montano et al., 2009). Due to random distribution of spikes during time, the sympathetic afferent can be expressed in terms of phasic and tonic activities, thus reflecting the dynamic integration of the reflex mechanisms controlling cardiovascular functions. The tonic and phasic activities of cardiovascular variabilities are coupled and synchronized in healthy individuals. However, HFrEF patients with NYHA functional classes III and/or IV can have a paradoxical pattern of sympathetic outflow, with a marked increase in tonus in the absence of low frequency oscillations, not only in heart rate variability, but also in sympathetic nerve variability (van de Borne et al., 1997). Thus, the interpretation of oscillatory patterns of sympathetic activity, together with the analysis of heart rate and arterial pressure variability's, could shed new light on the saturation of the autonomic nervous system; not only in HFrEF patients, but also in those suffering from HFpEF or HFmrEF patients. Another aspect that warrants further investigation is the interpretation of both codes. More specifically, an additional approach should be envisioned to obtain a distinctive profile of autonomic nervous control on the effector organ (autonomic modulation), or put differently, on the ability of autonomic nervous system to induce variations (i.e., heart rate and/or arterial pressure). The clinical significance of this ability is not yet completely understood, but it appears to have clinically relevant implications. In contrast to HFrEF, no clinical trials have documented an effective treatment for patients with HFmrEF and/or HFpEF. Therefore, due to the new classifications of HF, revisiting the autonomic reflexes and hyperadrenergic state in these new categories of HF patients could certainly offer new angles into the exploration of autonomic nervous system and therapeutic perspectives for these patients.

## Author contributions

All authors contributed to the preparation, revision and approval of the final manuscript.

### Conflict of interest statement

The authors declare that the research was conducted in the absence of any commercial or financial relationships that could be construed as a potential conflict of interest.
